# Executive functions and selective attention are favored in middle-aged healthy women carriers of the Val/Val genotype of the catechol-o-methyltransferase gene: a behavioral genetic study

**DOI:** 10.1186/1744-9081-6-67

**Published:** 2010-10-29

**Authors:** Silvia Solís-Ortiz, Elva Pérez-Luque, Lisette Morado-Crespo, Mayra Gutiérrez-Muñoz

**Affiliations:** 1 Departamento de Ciencias Médicas, División de Ciencias de la Salud, Campus León, Universidad de Guanajuato, León 37320, Guanajuato, México

## Abstract

**Background:**

Cognitive deficits such as poor memory, the inability to concentrate, deficits in abstract reasoning, attention and set-shifting flexibility have been reported in middle-aged women. It has been suggested that cognitive decline may be due to several factors which include hormonal changes, individual differences, normal processes of aging and age-related changes in dopaminergic neurotransmission. Catechol-O-methyltransferase (COMT), a common functional polymorphism, has been related to executive performance in young healthy volunteers, old subjects and schizophrenia patients. The effect of this polymorphism on cognitive function in middle-aged healthy women is not well known. The aim of the current study was to investigate whether measures of executive function, sustained attention, selective attention and verbal fluency would be different depending on the COMT genotype and task demand.

**Method:**

We genotyped 74 middle-aged healthy women (48 to 65 years old) for the COMT Val^158^Met polymorphism. We analyzed the effects of this polymorphism on executive functions (Wisconsin Card Sorting Test), selective attention (Stroop test), sustained attention (Continuous Performance Test) and word generation (Verbal Fluency test), which are cognitive functions that involve the frontal lobe.

**Results:**

There were 27 women with the Val/Val COMT genotype, 15 with the Met/Met genotype, and 32 with the Val/Met genotype. Women carriers of the Val/Val genotype performed better in executive functions, as indicated by a lower number of errors committed in comparison with the Met/Met or Val/Met groups. The correct responses on selective attention were higher in the Val/Val group, and the number of errors committed was higher in the Met/Met group during the incongruence trial in comparison with the Val/Val group. Performance on sustained attention and the number of words generated did not show significant differences between the three genotypes.

**Conclusion:**

These findings indicate that middle-aged women carriers of the Val^158 ^allele, associated with high-activity COMT, showed significant advantage over Met allele in executive processes and cognitive flexibility. These results may help to explain, at least in part, individual differences in cognitive decline in middle-aged women with dopamine-related genes.

## Background

Several studies have reported cognitive deficits such as poor memory, inability to concentrate, deficits in abstract reasoning, attention and set-shifting flexibility in middle-aged women [1-3]. It has been suggested that cognitive decline associated with age may be due to several factors which include hormonal changes [4,5], normal processes of aging [6,7], age-related changes in dopaminergic neurotransmission [8] and interindividual variations in brain function and cognitive abilities associated with genetic factors [9,10]. The findings from observational studies that have tried to link hormone levels and cognitive function in middle-aged women are inconclusive. Some studies report harmful associations, some protective and many fail to identify a clinically meaningful association between serum estrogen levels and cognitive ability [5]. These discrepancies may be due to methodological problems, such as failure to match groups on basic demographic characteristics, inadequate exclusionary criteria, the tests employed to assess cognition and insufficient control for affective disturbances are probably responsible, at least in part, for some of the contradictory findings [2,5]. Moreover, most of these studies have not considered genetic individual differences [11] and the genes involved in cognition [12]. Therefore, a behavioral genetic approach may help to explain, at least in part, the cognitive decline associated with age in middle-aged women, particularly in the modulation of the cognitive functions mediated by the prefrontal cortex (PFC) [7]. The study of genetic polymorphisms related to cognition provides a useful tool to investigate the functional role of genes expressed in the brain. One widely used approach is to relate allelic variants for functional measures at a biochemical or behavioral level, which has helped explain some cognitive deficits associated with age [8].

Evidence from animal and human models indicates that dopamine DA impacts on PFC function in accordance with an inverted U-shaped dose-response curve, such that the response is optimized within a narrow range of DA activity, with too little or too much DA having a relatively deleterious effect [13,14]. The DA system shows a marked decline with increasing adult age, with a gradual loss of both pre- and post-synaptic markers of DA neurotransmission from early through late adulthood [15]. This loss has been found in striatal, frontal and limbic areas [8], brain regions involved in learning and memory.

Some of the cognitive deficits reported in middle-aged women are functions modulated by the PFC [16], suggesting a role for DA system and their genes involved [17]. DA neurotransmission has been shown in both human and nonhuman primates to be a critical for cognitive functions subserved by PFC, such as executive cognition and working memory [18]. DA levels in the PFC are determined by DA biosynthesis and release and by the rate of diffusion, reuptake and degradation [19]. Catechol-O-methyltransferase (COMT) is the major mammalian enzyme involved in the metabolic degradation of released dopamine and accounts for more than 60% of this degradation in the frontal cortex [20]. The gene that encodes the COMT enzyme may influence cognition through its effects on dopaminergic function. The human COMT gene contains a functional polymorphism in the coding sequence (a G to A substitution), resulting in a valine (Val) to methionine (Met) substitution at codon 158 (Val^158^Met) that affects the thermostability of the enzyme [21]. As a result of the allelic differences in enzymatic activity, Val carriers have less DA activity in prefrontal cortex, while the Met/Met polymorphism produces a less active enzyme, resulting in higher dopamine levels than the Val/Val or the Val/Met polymorphism. Heterozygotes Val/Met show intermediate enzyme activity [22,23].

Studies in peripheral blood and in liver indicate that this functional polymorphism accounts for most of the human variation in peripheral COMT activity. Therefore, COMT genotype might also contribute to differences in prefrontal function between individuals [24]. The effect of the COMT genotype on cognitive functions related to the prefrontal cortex has been reported in several studies of healthy volunteers and psychiatric patients in mixed populations. In schizophrenic populations, as well as in normally functioning young adults, the Met/Met form of the COMT polymorphism has been related to better performance on a number of tests of executive function including the Wisconsin Card Sorting Test [23,25] and the n-back task [26]. In addition, carriers of the Met allele of COMT may elicit higher or lower levels of activity in the prefrontal cortex depending on the task characteristics and cognitive demands [27,28]. It has also been reported that older COMT Val homozygotes showed low levels of performance in executive functions if they were also BDNF Met carriers [29]. However, some studies, including a recent meta-analysis, have reported that the Met/Met form of the COMT polymorphism is not always associated with more efficient cognitive function or prefrontal activity compared with Val carriers [30-32]. Studies in non-demented older adults and healthy men aged 18-60 years have found better cognitive performance in Met homozygous individuals as compared to carriers of the Val allele [17,33,34], while other studies found no association between the COMT polymorphism and cognitive function [10,35,36]. Apparently, not all studies detected statistically significant differences between the three COMT genotypes-based groups, suggesting that the effects are relatively small and/or population-specific [37].

The effect of the COMT genotype on prefrontal functions in middle-aged healthy women is not well known. One feature of women over 50 years is a decrease in serum levels of estrogens that produces significant physiological effects [38]. There is a role for estrogen in cognitive functioning [39] and an influence on dopaminergic function in striatum [40]. Estradiol is synthesized in the brain via steroidogenic enzymes localized in the brain [41]. Estrogen functions as a multipurpose brain messenger that can interact with neurotransmitter systems at critical brain nuclei and facilitate neuronal function via gene expression and transmitter-gated ion channels. Estrogen action is mediated through estrogen receptors α and β, which are widely distributed throughout the brain and located in regions associated with cognitive functions [39]. Receptors for estrogen have been localized in the prefrontal cortex [42], and have been considered to this region of the brain as the site of estrogen's effect on cognition [43]. It has also been reported that estrogen is a regulator of COMT promoter activity. There are two estrogen response elements in the COMT promoter and that estrogen at physiological concentrations inhibits COMT mRNA expression in cells expressing estrogen receptors [44]. The estrogen-mediated decrease in COMT mRNA is accompanied by a decrease in COMT activity [45]. This inhibitory regulation by estrogens is consistent with evidence that women with high estrogen states have higher COMT activity than other women with low levels of estrogens [46]. One study reported that basal estrogen levels in postmenopausal women were similar between COMT genotypes. After administration of estrogens, women carriers of the COMT^LL ^genotype, with low COMT activity, increased estrogen levels [47]. Moreover, compared with men, women have higher striatal [^18 ^F] fluorodopa uptake, suggestive of greater presynaptic dopamine synthesis [48], a lower D2 receptor affinity that reflects higher dopamine levels [49] and a greater dopamine transporter uptake [50]. However, estrogenic state (menopause or menstrual cycle) has not been fully taken into account in many cognitive studies of COMT activity and may be a significant confounder [51].

DA mechanisms may be particularly relevant in prefrontal function in women with low levels of estrogen in middle age. Women with certain genetic makeup may respond differently to prefrontal task either facilitating or impairing cognitive performance. It has been postulated that the COMT Met allele, associated with low enzyme activity, is of benefit during tasks of cognitive stability requiring tonic dopamine activation, but detrimental on tasks of cognitive flexibility requiring phasic activation [52]. These effects have been found in heterogeneous groups, but it is not well known whether these effects might occur in the same way in a homogeneous group of middle-aged women with low estrogen levels. The aim of the current study was to examine the effects of the COMT Val^158^Met genotype on the response to neuropsychological tests that demand prefrontal functions in middle-aged healthy women. It was hypothesized that measures of executive functions, selective attention, sustained attention, and verbal fluency would be different depending on the COMT genotype and task demand. In the present study, a sample of middle-aged healthy women was genotyped for the COMT Val^158^Met polymorphism to analyze the effect on cognitive performance. An advantage of the Val allele over Met carriers only in tests that demand more resources in executive processes and cognitive flexibility was found, but not in tests that demand sustained processes or verbal fluency. These findings suggest that some prefrontal functions demanding cognitive flexibility are favored in women carriers of the Val allele, which may help to explain, at least in part, the cognitive decline in middle-aged women with dopamine-related genes.

## Methods

### Subjects

Seventy-four middle-aged healthy postmenopausal women volunteers between 48 and 65 years old with an intact uterus were genotyped for the COMT Val^158^Met polymorphism in a crossover design. The sample size of 74 women with the Val^158^Met polymorphism was calculated to yield an expected power of 0.86 to detect a difference of 10% on cognitive task performance with a two-sided significance level of α = 0.05. All women underwent a medical interview to assess their health status. Women had been amenorrheic for at least 12 months, with no history of cardiovascular, metabolic, endocrinological or malignant diseases. None of them was on any type of medication or had ever received hormonal treatment. Incipient dementia was ruled out using the Mini-Mental State Examination (MMSE) [53]. The scores of this test range from 0 to 30, and subjects with dementia generally score below 24. In the present study groups, the MMSE scores ranged from 27 to 30. Each woman was tested in one session by one trained female investigator during the same time of day (between 0900 h and 1100 h). This study was approved by the Ethics Committee of the Department of the Medical Sciences of the University of Guanajuato, and all of the women provided their written informed consent.

### Genotyping

To detect the Val^158^Met polymorphism in the COMT gene, genomic DNA was extracted from peripheral blood leukocytes using standard methods. The portion of exon 4 that contains the polymorphic site was amplified by PCR in a total reaction volume of 27 μl containing 25 pmol of forward primer 5'-TACTGTGGCTACTCAGCTGTGC-3' and reverse primer 5'-GTGAACGTGGTGTGAACACC -3', 100 ng genomic DNA, 2 mM of MgCl_2_, and 250 μM dNTPs. PCR conditions were as follows: denaturation at 94°C for 1 min, and 30 cycles of denaturation (94°C, 30 sec), annealing (56°C, 30 sec), and extension (72°C, 30 sec), and a final extension at 72°C for 10 min. The PCR products were digested with Hsp92ll (Promega) at 37°C overnight and electrophoresed in a 4% agarose gel and stained with ethidium bromide. The expected products after digestion were Val/Val homozygote (114 bp), Val/Met heterozygote (114 bp, and 96 bp), and Met/Met homozygote (96 bp) [47].

### Neuropsychological Tests

Four standard neuropsychological tests that measure prefrontal functions [54] were used to evaluate the effects of the COMT Val^158^Met polymorphism in middle-aged women. Each test was administered during one session and was distributed at random. The neuropsychological tests are described below according to the abilities they represent.

### Executive functions

The Wisconsin Card Sorting Test (WCST) [55] was used to evaluate executive functions of the prefrontal cortex. The performance of the WCST produces physiological activation in a network of regions including the dorsolateral prefrontal cortex, the inferior parietal lobule, and the posterior portion of the inferior temporal cortex [56]. The WCST is an abstract reasoning and problem solving test that involves the use of working memory to form a cognitive set and apply a conceptual strategy but also that necessitates maintenance and then shifting of the set when appropriate. The stimuli were shown on a screen facing the subjects. The WCST requires subjects to discover the principle under which a deck of cards must be sorted. The standard material consists of cards bearing geometric figures that vary in color (red, green, blue, or yellow), form (triangle, star, cross, or circle) and number (1, 2, 3 or 4 items). Four reference cards are aligned in front of the subject throughout the test. Another deck of cards serves as response cards. The subject is instructed to place each response card in front of 1 of the 4 reference cards, wherever she thinks it should go. After each response, she is told whether the response was "right" or "wrong" but not where the card should have gone. The subject's goal is to obtain as many "right" responses as possible. Initially, cards must be sorted according to color. When performance is successful, the sorting rule is changed, from color to form or number; the subject must recognize the change and discover the new correct rule. The following results provided by the WCST in a computerized version were analyzed: (1) the number of categories completed, refer to effectiveness of measured cognitive function; (2) the number of correct responses (trials), taking into account the ability to reach successful or unsuccessful outcomes; (3) perseverative errors refer to the number of errors committed by the subject by pursuing an criterion which has received the information that is not valid; (4) errors refer to the number of incorrect answers. We examined these measures because they are the most commonly used to evaluate the WCST performance [17].

### Selective attention

A computerized version of the Stroop test was used to examine selective attention ability [57]. Performance of this test activates the frontal and cingulate cortices [58]. This test entails multiple cognitive processes including selective attention, response inhibition, interference control, and response speed. The test requires that subjects rapidly shift the perceptual set when viewing the names of colors that appear in matching or non-matching colors. The test consists of a sequence of words that denote the colors green, blue and red and that are displayed in the same colors on the screen. The subjects were instructed to perform the task in two trials. In the first trial, the subject should ignore the text color and press the ← button if the GREEN word appears the ↓ button for the BLUE word, and the → button for the RED word. In the second trial, the subject should ignore the meanings of the word and press the same buttons according to text color. Reaction time, number of correct responses, number of no responses and number of errors were computed.

### Sustained attention

A computerized version of the Continuous Performance Test (CPT) was used to examine the ability to sustain attention [59]. Performance of CPT activates the right frontal and parietal lobes [60]. The sustained attention test consists of 150 alphabet letters displayed continuously in a random sequence, one at a time, for 50 milliseconds on a video screen. The inter-trial interval ranged randomly from 5 to 7 seconds. The subjects were instructed to perform the test with two different levels of difficulty. In the first level, the letter "S" pattern was selected as the target stimulus, and the subject was asked to press the "enter" button on the keyboard as soon as possible each time the target stimulus was perceived. In the second level, this instruction was maintained, and the subject was asked to press the "enter" button only when the target stimulus was preceded by a specified item, the letter "A". Reaction time, omissions, errors and correct responses were computed.

### Verbal fluency

The Word Fluency Test [61] was used to evaluate the spontaneous generation of words according to an initial given letter. This test reflects function in the left frontal lobes [62]. The word production test consisted of asking subjects to write as many words as they could that begin with the alphabet letters F, L, and M, excluding proper nouns, numbers, and the same word with a different suffix, in 1 min. The score was the sum of three 1 min trials with different letters.

### Hormonal measurements

A blood sample of 10 ml was extracted from participants to determinate the hormonal levels of 17β-estradiol and progesterone by the ELISA method, and LH and FSH by radioimmunoassay using commercial kits. The serum levels of these hormones corroborated the hormonal status of the postmenopausal women.

### Statistical analysis

Statistical analysis was performed with STATISTICA for Windows 8 (StatSoft, Inc). Data were tested for a normal distribution using Levene's test before statistical procedures were applied. Kruskal-Wallis one-way analysis of variance (ANOVA) was used to compare the demographic characteristics between the Val/Val, Val/Met and Met/Met COMT genotypes. All measures from different neuropsychological test scores were converted to z-scores to compare the values between the three genotype groups. This statistical tool produces new variables with a standardized value, and it describes the location of the value in terms of the standard deviation relative to the mean [63]. A separate one-way analysis of variance (ANOVA) was computed for each test to compare the z-scores between the Val/Val, Val/Met and Met/Met COMT genotypes. The analysis included age and education as covariates. The Visual Statistics System (ViSta) for Windows 7.9 module "Effect size" was used to correct the data outlier and estimate the effect sizes. Effect sizes are indicated by the coefficient (ρ_1_) between groups. The ρ_1 _values were squared to ease interpretation in terms of the percentage of the total variance associated with an effect. Differences were considered as significant with alpha levels of p < 0.05. A post-hoc comparison was performed using Tukey's test. In the present study, we only analyzed one specific locus and, therefore, it is not necessary to correct the P-value for multiple testing [64].

## Results

### Characteristics of COMT genotypes

Genetic analysis identified 27 middle-aged women with Val/Val COMT genotype, 15 with the Met/Met genotype, and 32 with the Val/Met genotype, a distribution consistent with the Hardy-Weinberg equilibrium (X^2 ^= 0.946). The demographic and clinical characteristics of these genotypes are shown in Table 1. The three genotypic groups did not differ significantly in age, schooling, menarche, menopausal years, blood pressure, weight, height, body mass, pregnancies or hormonal levels of FSH, LH, 17β-estradiol, and progesterone.

**Table 1:** Middle-aged woman characteristics

	Val/Val (N = 27)	Val/Met (N = 32)	Met/Met (N = 15)	H	p*
Age (years)	53 (48-64)	53 (48-51)	58 (51-65)	5.28	0.07
Years of education	9 (6-15)	9 (6-15)	6 (6-11)	0.28	0.50
Menarche (years)	13 (11-16)	13 (10-18)	13 (10-14)	1.22	0.24
Menopause (years)	46 (31-56)	48 (35-56)	48 (35-57)	1.68	0.10
Partus	3 (0-7)	3 (0-11)	5 (0-9)	0.50	0.77
Height (cm)	156 (146-164)	156 (145-162)	155 (140-177)	1.85	0.39
BMI (kg/m2)	27.8 (20.57-40.34)	28.15 (23.50-38.44)	29.35 (20.78-38.44)	0.56	0.75
TAS (mmHg)	110 (90-14)	108 (88-140)	110 (100-14)	1.92	0.38
TAD (mmHg)	80 (60-90)	80 (60-90)	80 (70-90)	1.14	0.92
FSH (mIU/ml)	61.25 (32.1-134.8)	56.5 (38.6-111.9)	57.15 (32.2-110.3)	0.49	0.82
LH (mIU/ml)	21.25 (9.1-64.2)	22.75 (10-37.6)	14.7 (10.2-61.9)	1.25	0.82
E (ng/ml)	14.95 (2-26.7)	14 (1-21.5)	12.45 (5.3-14.2)	0.28	0.45
Progesterona (ng/ml)	0.5 (0.4-1)	0.33 (0.1-1.2)	0.5 (0.1-1.5)	2.00	0.18

Values are shown as the median and range

*Kruskal-Wallis test

BMI = Body Mass Index

TAS = Systolic arterial tension

TAD = Diastolic arterial tension

E = 17β-estradiol

### Neuropsychological performance

#### Executive functions: WCST

**Figure 1:**
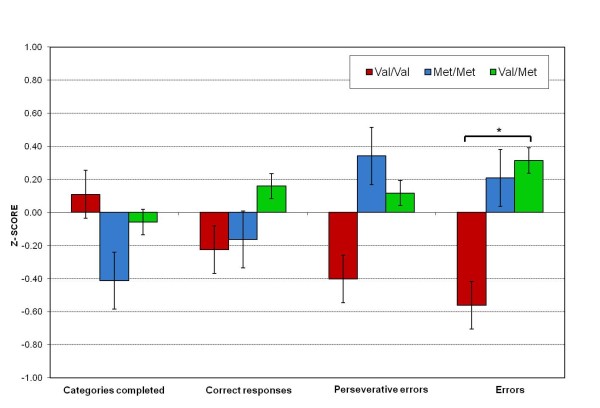
**shows the z-scores of executive functions (Wisconsin Card Sorting Test) for the Val/Val, Met/Met and Val/Met genotypes**. Asterisks indicate significant differences between the genotypes (*p < 0.05).

The results of executive functions scores from WCST variables transformed to z-scores for the three genotypic groups are shown in Figure 1. The number of categories completed (F = 0.46, p = 0.96), the correct responses (trials), taking into account the ability to reach successful or unsuccessful outcomes (F = 0.62, p = 0.31) and perseverative errors (F = 1.87, p = 0.36) were not significant between the three genotype groups. However, the decrease in perseverative errors in the Val/Val genotype group was marginally significantly (β = -0.37, p = 0.05). The main effect was significant for the total of errors committed (F = 3.22, p = 0.02, ρ_1 _= 0.38, explaining 14.4% of the total variance in the data). The z-score denoted that women carriers of the Val/Val genotype performed better, as indicated by a lower number of total errors committed below the mean (M = -0.56, SE = 0.22), than the Val/Met (M = 0.31, SE = 0.30) or the Met/Met (M = 0.21, SE = 0.15) groups, both of which committed errors above the mean. The post-hoc comparison between the Val/Val and Val/Met genotypes was significant (p = 0.04). The decrease in total errors in the Val/Val genotype group was significant (β = -0.47, p = 0.01).

#### Selective attention: Stroop test

**Figure 2:**
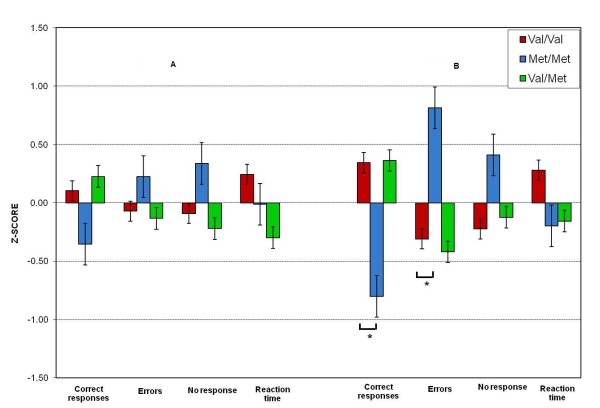
**shows the z-scores of selective attention (Stroop test) during the congruence trial in A and incongruence trial in B for the Val/Val, Met/Met and Val/Met genotypes**. Asterisks indicate significant differences between the genotypes (*p < 0.05).

The results of selective attention scores from Stroop test variables transformed to z-scores for the three genotypic groups are shown in Figure 2. There was no significant difference between the COMT genotypes in the first level of difficulty for the congruence trial on selective attention test. The number of correct responses (F = 1.11, p = 0.33), the number of errors committed (F = 0.41, p = 0.66), the number of no response (F = 1.00, p = 0.37) and reaction time (F = 0.957, p = 0.39) were similar among the genotypes. The main effect for COMT on the selective attention test was significant for the number of correct responses (F = 7.34, p = 0.002, ρ_1 _= 0.57, explaining 32.4% of the total variance in the data), and the number of errors made (F = 7.74, p = 0.001, ρ_1 _= 0.53, explaining 28% of the total variance in the data) during the incongruence trial. Women carriers of the Val/Val genotype performed better, as indicated by a higher number of correct responses above the mean (M = 0.34, SE = 0.19), than the Met/Met genotype, which performed below the mean (M = -0.80, SE = 0.29). Women with the Val/Val genotype also committed a lower number of errors below the mean (M = -0.30, SE = 0.19) than did the Met/Met genotype, which performed above the mean (M = 0.81, SE = 0.33). Post-hoc analysis revealed significant differences for the number of the correct responses between the Val/Val and Met/Met groups (p = 0.005) and Val/Met versus Met/Met (p = 0.006), as well as for number of errors committed between the Val/Val and Met/Met (p = 0.005) and Val/Met versus Met/Met groups (p = 0.003). The decrease in the number of correct responses made by the Met/Met genotype during the incongruence trial was significant (β = -0.63, p = 0004), while the increase in these responses observed in the Val/Val group was significant (β = 0.33, p = 0.04). The increase in the number of errors committed by the Met/Met genotype was significant (β = 0.63, p = 0.0004).

#### Sustained attention: CPT

The ANOVA analysis did not reveal a significant difference for any variable of the sustained attention test. In the first difficulty level, the number of correct responses (F = 0.747, p = 0.48), the number of errors (F = 0.539, p = 0.58), the number of omissions (F = 0.273, p = 0.76), and the reaction time (F = 1.355, p = 0.27) were similar among the three genotypes. During the second difficulty level, the number of correct responses (F = 1.726, p = 0.19), the number of errors (F = 0.366, p = 0.69), the number of omissions (F = 1.591, p = 0.21), and the reaction time (F = 2.167, p = 0.13) were also similar among the three genotypes.

#### Verbal fluency

The ANOVA analysis did not show significant differences for the number of words generated on the verbal fluency test (F = 0.369, p = 0.69) among the three genotypes.

## Discussion

As hypothesized the measures of executive functions, selective attention, sustained attention, and verbal fluency were different depending on the COMT genotype and task demand. Women carriers of the Val/Val genotype showed an advantage over the Met/Met or Val/Met genotypes in executive functions, as indicated by the fewer number of errors committed on the WCST. This result explained 14.4% of the total variance. The Val/Val group also showed an advantage over the Met/Met genotype on selective attention, as indicated by the greater number of correct responses and lower number of errors made during the incongruent trial. These results explained 32.4% and the 28% of the total variance, respectively. Interestingly, an effect of the COMT genotype was not found on sustained attention and words generation performance, as measured by the Continuous Performance Test and Word Fluency test, respectively. It was also interesting to note that women carriers of the Val allele performed better only in tests that demand more resources in cognitive flexibility and working memory, but not in tests that demand sustained processes and words generation. Other studies in heterogeneous groups have found different effects of the COMT genotype. Some behavioral studies using tasks that involve executive functioning (such as WCST) or working memory tasks (such as n-back) have found an advantage of Met over Val carriers among normally functioning young adults [23,65], in a mixed sample of young adults and middle-aged women and men [31], in adult and elderly men [34], and in schizophrenic populations [25]. These effects have been found on the performance tasks that demand sustained processes such as maintaining a cognitive set, but not in processes related to cognitive flexibility.

The results of present study are supported by the tonic and phasic dopamine theory and its relation with the COMT polymorphism. It has been hypothesized that the Val allele, which is associated with high activity COMT, increases phasic and reduces tonic dopamine transmission subcortically and decreases dopamine concentrations cortically. This leads to an increase in D_2 _and a decrease in D_1 _transmission. As a result, there is decreased stability of neural networks underlying working memory representations, including those that are responsible for the maintenance of executive functions. However, this phenomenon also facilitates the switching to novel tasks or transitions to alternate network states that mediate the resetting of working memory traces benefitting on task demand flexibility [52]. The present study found that the middle-aged women carriers of the Val allele showed more benefit in tasks that demand executive functions and cognitive flexibility, presumably because an increased prefrontal dopamine levels during the tasks. This result suggests that these women had a greater capacity to sustain cognitive representations in working memory, which is crucial for executive functions. Biophysical models of the prefrontal cortex neuronal architecture suggest that sustained D_1 _activation (tonic dopamine) helps establish and maintain the stability of neural networks by preventing uncontrolled, spontaneous switches into high activity states (i.e., spontaneous activation of task-irrelevant representations) [66].

It has also been hypothesized that the Met allele, which is associated with low activity COMT, decreases phasic and increases tonic dopamine transmission subcortically and increases dopamine concentrations cortically. These phenomena are associated with increased D_1 _and decreased D_2 _transmission in the prefrontal cortex. This increases the stability of networks that mediate sustained working memory representations and benefits task demand stability and sustained processes, but it may show excessive cognitive rigidity [52]. The present study found that the middle-aged women carriers of the Met allele showed less benefit on tasks that demand executive functions and cognitive flexibility. This result suggests that these women had more difficulty switching or updating the currently active networks that represent sustained working memory representations. They had difficulty choosing the appropriate response to external change, which resulted in excessive repetition of maladaptive behaviors, perseveration, stereotypy and a failure to detect novelty. Interestingly, the present study found no effect of the COMT genotype on sustained attention and words generation performance, as measured by the Continuous Performance Test and Word Fluency test, respectively. These findings suggest that middle-aged women are able to maintain the prior stimulus traces over time as well as word generation with a specific letter of the alphabet. In addition, they did not require more cognitive resources to be successful, which could be influenced by the activation of tonic dopamine [52].

The findings of present study are consistent with the neural and cellular mechanisms described for prefrontal functions. Neural correlates have suggested that the effects of DA on cognition and brain activity seem to be modified by COMT genotype and task demand. A study showed that amphetamine administration to healthy young subjects Val carriers (low endogenous levels) enhanced the efficiency of prefrontal cortex function during a N-back working memory task. In contrast, Met carriers (high endogenous levels) showed less efficient frontal response and the drug caused deterioration at high working memory load. It was also found that individuals with the Val/Val genotype perform better on amphetamine (fewer errors), whereas individuals with the Met/Met genotype get worse (more errors) during the WCST performance [14]. The cellular mechanisms have provided information about how the DA modulates PFC function. Some studies suggest that DA strengthens the effects of strong depolarizing currents and enhances task-related neural activity [67] through the activation of D1 receptors, which enhances persistent NA^+^, L-type Ca^2+^, and *N*-methyl-D- aspartate currents in PFC pyramidal neurons [68]. The net result of D1 receptor stimulation is signal sharpening, or a gain-amplifying effects on a subset of inputs to PFC neurons [69]. Evidence also indicates that too much DA activity in the PFC may disorganize networks of PFC neurons by activating inhibitory mechanisms, including inactivation of N-type Ca^2+ ^channels, activation of GABAergic interneurons and pre- and postsynaptic reduction of glutamate-mediated synaptic responses [70].

In the current paper, a group of women was studied in the post-menopausal period, which is characterized by a decrease of estrogens [38]. Significant differences in hormone levels among the genotypes were not found. These results are consistent with other studies that have found similar concentrations of 17β-estradiol between COMT genotype [47], low estradiol levels in postmenopausal women with the Val/Val genotype [71] and high concentrations of urinary 2-hydroxyestrone in women with Met/Met genotype [72]. Behavioral studies have shown that low levels of estrogen seem to facilitate certain cognitive function in postmenopausal women. One study found that women with lower estrone levels had significantly better performance on Digit Symbol compared with women with higher estrone levels [73]. The neurophysiological mechanisms involved in this process are not well known and are probably related to the role of estrogens in the regulation of COMT activity [44]. In addition, other genetic polymorphisms involved in the metabolism of estrogens that may influence sex hormones concentrations have been identified. It has been reported that carrying two CYP19 7r(-3) alleles (gene encodes aromatase) had lower estrone and estradiol concentrations compared with noncarriers. Women carriers at least one CYP19 8r allele had higher estrone, higher estradiol and higher free estradiol concentrations compared with noncarriers [72]. The influence of these polymorphisms may explain, at least in part, the similarity in the levels of estrogen found in present study. Future research is required to elucidate the relationships among COMT genotypes, hormonal status and cognitive performance.

It is also important to note that the middle-aged women analyzed in the present study were healthy volunteers without any apparent history of hyperactivity disorder, anxiety or depression. However, the findings of the present study seem to indicate that women carrying the Met allele showed indices of inattention, a lack of inhibitory control, impulsivity and poor working memory, which appear to be consistent with the dynamic developmental theory of attention-deficit/hyperactivity disorder [74]. Some studies have found that the Met allele of the COMT Val158Met is associated with increased ADHD symptom severity in children [75], girls with ADHD were more likely than boys to have the predominantly inattentive type of ADHD [76], anxiety in women [77] and with lower extraversion personality and neuroticism among healthy adults of both genders [78]. Future research should consider previous history of this disorder in adults included in behavioral genetic studies, particularly in studies with women.

The current results add new information concerning to influence of DA mechanisms on prefrontal functions whose beneficial effects are associated with dopamine-related genes in a homogeneous group of middle-aged women with low estrogen levels. According to the results of present study, an advantage of the Val allele over Met carriers only in tests that demand executive processes and cognitive flexibility was found, but not in tests that demand sustained processes or verbal fluency. These findings suggest that the prefrontal functions show a benefit depending on the COMT genotype and task demand, which partially explains, individual differences in cognitive decline in middle-aged women. The results of this study provide important new leads into the complex relationships between genes and prefrontal functions and may contribute to a better understanding cognitive function associated with age in healthy women.

## Limitations

There are some limitations that must be addressed in the present study. This study was conducted in a specific sample of Mexican middle aged-women with low levels of estrogen during the postmenopause. The study did not include women with high estrogen levels, which must be included in future approaches to determine whether there are differences associated with the COMT gene. It also must include the analysis of other polymorphisms associated with estrogen metabolism. Another limitation of the study is that only included a group of women between 48 and 65. Other studies must include individuals of different ages and gender.

## Conclusions

In conclusion, an advantage of Val over Met carriers among normally functioning middle-aged women with low estrogen levels only in tests that demand executive processes and cognitive flexibility was found, but not in tests that demand sustained processes or verbal fluency. These findings suggest that the prefrontal function show a benefit depending on the COMT genotype and task demand, which partially explains, individual differences in cognitive decline in middle-aged women with dopamine-related genes.

## Competing interests

The authors declare that they have no competing interests.

## Authors' contributions

SSO conceived and designed the study. MGM and LMC are graduate students. MGM and SSO performed the study. EPL and MGM carried out the molecular genetics analysis. MGM and LMC participated in the evaluation of the subjects. MGM and SSO performed the data analysis. MGM helped to draft the manuscript. SSO drafted the manuscript. All authors read and approved the final manuscript.
